# Identification of South African Plant-Based Bioactive Compounds as Potential Inhibitors against the SARS-CoV-2 Receptor

**DOI:** 10.3390/ph17070821

**Published:** 2024-06-22

**Authors:** Nqobile Monate Mkolo, Clarissa Marcelle Naidoo, Rose Kadye, Chikwelu Lawrence Obi, Benson Chucks Iweriebor, Oyinlola Oluwunmi Olaokun, Earl Prinsloo, Muhammad Sulaiman Zubair

**Affiliations:** 1 Department of Biology and Environmental Sciences, Sefako Makgatho Health Sciences University, Pretoria 0204, South Africa; nqobile.mkolo@smu.ac.za (N.M.M.); lawrence.obi@smu.ac.za (C.L.O.); benson.iweriebor@smu.ac.za (B.C.I.); oyinlola.olaokun@smu.ac.za (O.O.O.); 2 Department of Biotechnology, Rhodes University, Makhanda 6140, South Africa; rose.kadye@ru.ac.za (R.K.); e.prinsloo@ru.ac.za (E.P.); 3 Department of Pharmacy, University of Tadulako, Palu 94118, Indonesia; sulaimanzubair@untad.ac.id

**Keywords:** spike (S) glycoprotein, angiotensin-converting enzyme 2 (ACE2), *Artemisia annua*, *Artemisia afra*, tryptanthrin, 6″-O-acetylglycitin, 25-hydroxyvitamin D3-26,23-lactone, sesaminol glucoside, anti-COVID bioactive compounds

## Abstract

The expected progress in SARS-CoV-2 vaccinations, as anticipated in 2020 and 2021, has fallen short, exacerbating global disparities due to a lack of universally recognized “safe and effective” vaccines. This study focuses on extracts of South African medicinal plants, *Artemisia annua* and *Artemisia afra*, to identify metabolomic bioactive compounds inhibiting the binding of the SARS-CoV-2 spike protein to ACE2 receptors. The extracts were monitored for cytotoxicity using a resazurin cell viability assay and xCELLigence real-time cell analyzer. Chemical profiling was performed using UPLC-MS/MS, orthogonal projection to latent structures (OPLS), and evaluated using principle component analysis (PCA) models. Identified bioactive compounds were subjected to in vitro SARS-CoV-2 enzyme inhibition assay using standard methods and docked into the spike (S) glycoprotein of SARS-CoV-2 using Schrodinger^®^ suite followed by molecular dynamics simulation studies. Cell viability assays revealed non-toxic effects of extracts on HEK293T cells at lower concentrations. Chemical profiling identified 81 bioactive compounds, with compounds like 6″-O-acetylglycitin, 25-hydroxyvitamin D3-26,23-lactone, and sesaminol glucoside showing promising binding affinity. Molecular dynamics simulations suggested less stable binding, but in vitro studies demonstrated the ability of these compounds to interfere with SARS-CoV-2 spike protein’s binding to the human ACE2 receptor. Sesaminol glucoside emerged as the most effective inhibitor against this interaction. This study emphasizes the importance of multiplatform metabolite profiling and chemometrics to understand plant extract composition. This finding is of immense significance in terms of unravelling metabolomics bioactive compounds inhibiting the binding of the SARS-CoV-2 spike protein to ACE2 receptors and holds promise for phytotherapeutics against SARS-CoV-2.

## 1. Introduction

An extremely infectious severe acute respiratory disease, characterized by symptoms such as fever, fatigue, dry cough, and respiratory issues was initially reported in Wuhan, China in December 2019 [1]. Later, the World Health Organization (WHO) on 11 February 2020 formally classified this infection as the coronavirus disease 2019 (COVID-19), attributing it to the severe acute respiratory syndrome coronavirus 2 (SARS-CoV-2) virus [2,3,4,5,6,7].

The WHO officially declared the coronavirus outbreak a pandemic on 11 March 2020 [2,3,8]. Despite the pandemic no longer being categorized as a major health crisis, global populations continue to grapple with various health issues stemming from the persistent post-COVID syndrome, encompassing physical, cognitive, and immunosuppression deterioration [9,10,11]. Despite the knowledge surrounding the pathogenesis of the virus, it caused increased mortality and morbidity rates, especially in poorly developed countries with inadequate healthcare systems [12]. Efforts to mitigate the effects of COVID-19 and long-term COVID-19 and associated infections include antiviral and antimicrobial drugs, immunomodulators, and vaccines; however these are often limited in low-income countries [12,13,14]. The expectations projected in 2020 and 2021 regarding the progress of COVID-19 vaccinations have not been met [15]. Moreover, there is still work to ensure that vaccines are distributed fairly nationally and internationally, especially in impoverished nations. Global disparities in vaccination coverage have been exacerbated by the lack of “safe and effective” vaccinations [16,17]. Booster doses are often needed to strengthen immunity; however, only 41% of the South African population recently received at least one dose of vaccination [16,17,18]. Under these conditions, the necessity of a plant-based solution for the treatment of COVID-19 has emerged.

The transmembrane spike (S) glycoprotein, which plays a critical role during virus interaction with the human angiotensin-converting enzyme-2 (ACE2) receptor during cell entry, is considered a significant target in anti-COVID-19 drug development [1,3]. Bioactive compounds isolated from natural plant products are often used for the development of innovative pharmaceuticals [13], including possible agents against SAR-CoV-2 [12,14].

South African traditional medicinal plants have shown a diverse range of compounds with many biological activities including the treatment of viral diseases [19]. The medicinal plant *Artemisia annua* (L.), commonly known as sweet annie, belongs to the Asteraceae family and is described as an annual short-day plant, with a maximum height of 3 m. *A. annua* is distributed across several regions including North China, Europe, North Africa, North India, North Vietnam, USA, Argentina, and South Africa, where it often grows in hillsides, edges of forests, and in wastelands [20]. In southern Africa, the plant is often used traditionally for treating respiratory tract infections, fevers, and malaria [21]. Phytochemical screening of *A. annua* reveals that artemisinin and sesquiterpenes, caryophyllene oxide, caryophyllene, farnesene, and germacrene D are the most abundant chemicals in the oil [22]. These compounds may be responsible for their pharmacological potential as pharmacological reports of *A. annua* have shown anti-chloroquine-resistant, anti-cerebral malaria, and antimicrobial properties [21,23]. *Artemisia afra* (Jacq.), also known as African wormwood, is a naturalized South African medicinal plant also belonging to the Asteraceae family [24]. *A. afra* is well distributed in the mountain regions of South Africa, Namibia, Zimbabwe, Kenya, Tanzania, Uganda, and North Ethiopia [25,26]. Its pharmacological properties range from antibacterial, antiviral, and anti-inflammatory activities, with bioactive compounds such as sesquiterpenes, glaucolides, guaianolides, long-chain alkanes, organic acids, coumarins, flavonoids, and monoterpene [26,27]. Despite the wealth of information regarding the bioactive compounds of South African medicinal plants and their potential pharmacological benefits, including possible treatment options for SARS-CoV-2, investigations of South African medicinal plants as likely treatment agents for SARS-CoV-2 have not received the desired attention by researchers.

This study, therefore, focuses on two South African medicinal plants for the discovery of bioactive compounds displaying anti-SARS-CoV-2 activities. To address these concerns, the cytotoxicity of *A. annua* and *A. afra* extracts was tested, and advanced analytical techniques including ultra-high-performance liquid chromatography–tandem mass spectrometry (UPLC-MS/MS), molecular docking, and in vitro inhibition assays were used to identify the efficacy of these extracts and provide a rational phytotherapeutic choice to SARS-CoV-2.

## 2. Results

### 2.1. Resazurin Cell Viability Assay

**Figure 1:**
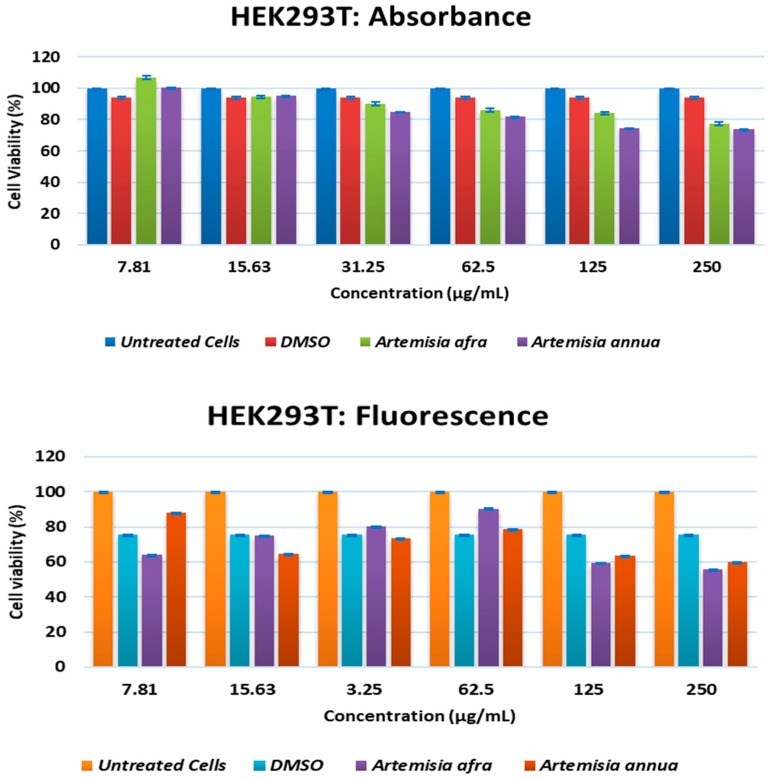
Viability of HEK293T cells after 24 h of treatment with *A. annua* and *A. afra* leaves’ extracts at different concentrations ranging from 250 to 7.81 µg/mL using absorbance for resorufin and fluorescence-based detection.

The extracts of *A. annua* and *A. afra* leaves were evaluated using the double serial dilution ranging from 250 µg/mL to 7.81 µg/mL for the resazurin cell viability assay. Human embryonic kidney 293 transformed (HEK293T) cells exhibited viability values of >77.23% and >73.82% after *A. afra* and *A. annua* treatment, respectively, after 24 h at different concentrations when the absorbance for resorufin was measured at 570 nm (Figure 1). However, fluorescence-based detection showed lower viability for HEK293T cells, with a value >55.89% viability after *A. afra* treatment and >59.87% after *A. annua* treatment at all different concentrations.

### 2.2. Cell Viability Assay Using xCELLigence Real-Time Cell Analyzer

**Figure 2:**
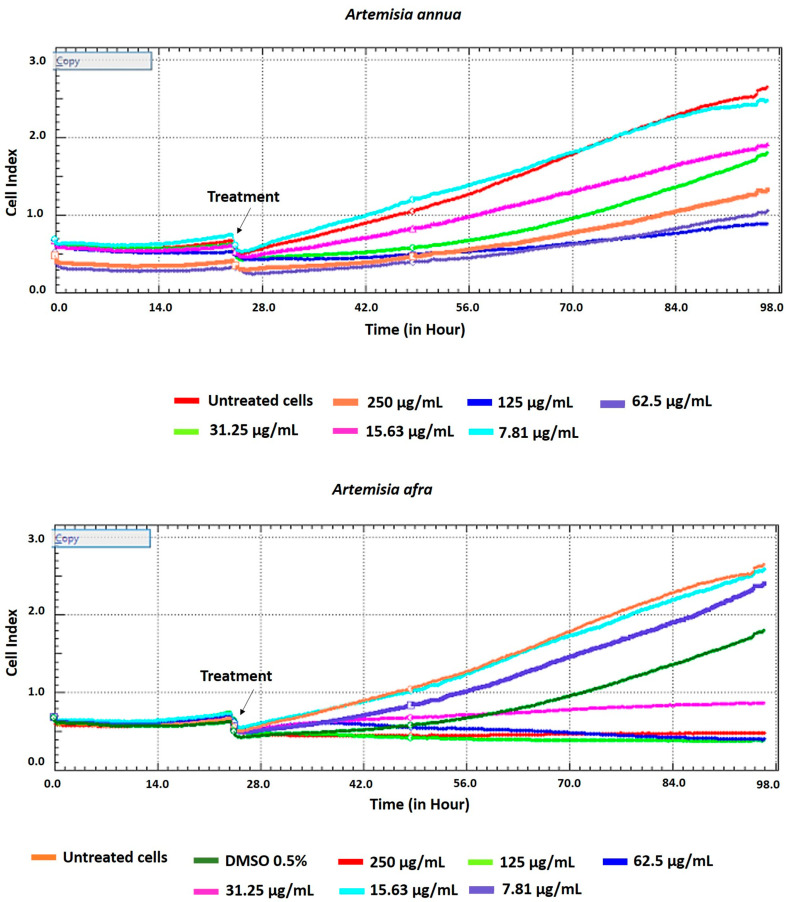
Real-time monitoring of HEK293T cells after treatment with *A. annua* and *A. afra* leaves’ extracts at different concentrations ranging from 250 to 7.81 µg/mL using the xCELLigence RTCA analyzer.

The viability of HEK293T cells after treatment with *A. annua* and *A. afra* leaves’ extracts was measured using the xCELLigence real-time cell analyzer (RTCA). The methanol extract of these plants showed no significant cytotoxic effect on the HEK293T cells at lower concentrations of 31.25, 15.63, and 7.81 µg/mL. Records from RTCA indicated a proliferative phase in the cell index (CI) values between 24 and 98 h post-treatment when lower concentrations (31.25, 15.63, and 7.81 µg/mL) of both plant extracts were used. An increase in CI values was observed for both the untreated and dimethyl sulfoxide (DMSO)-treated HEK293T cells (Figure 2).

### 2.3. Chemical Profiling of A. annua and A. afra Leaves

**Figure 3:**
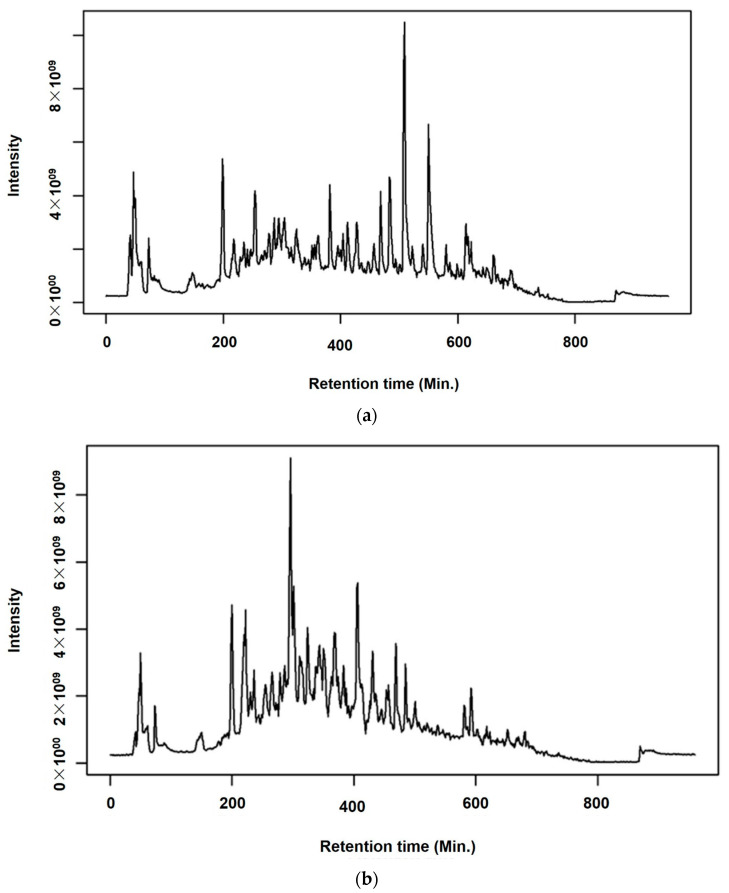
Base peak intensity (BPI) chromatograms obtained from the positive ion UPLC-MS/MS analyses of (**a**) *A. annua* and (**b**) *A. afra* samples.

**Figure 4:**
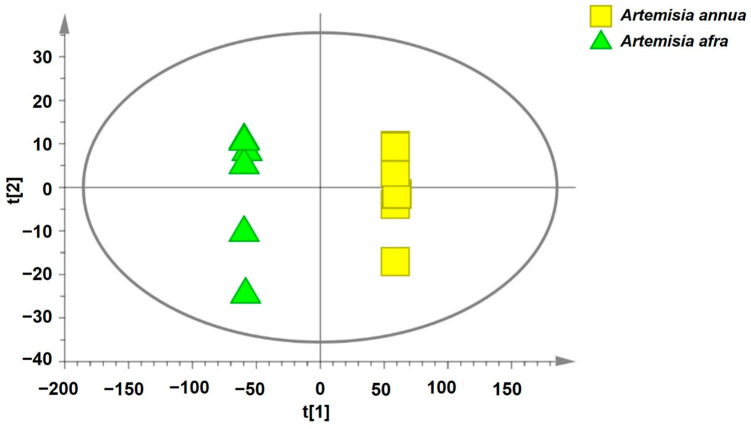
The scores scatter plot of the PCA model of *A. annua* and *A. afra* metabolite groups.

The base peak intensity (BPI) chromatograms of the sample in ESI+ mode from *A. annua* and *A. afra* leaves exhibited a total of 225 peaks (Figure 3). These identified compounds belong to different classes of secondary metabolites including triterpenoids, flavonoids, iridoid glycosides, hydroxy fatty acids, phenolic acids, carboxylic acid, phenylethanoid, and glycosides. Table S1 (Supplementary Material) presents the data for the identified compounds including retention times, molecular weights, *m*/*z* values, human metabolome database identification (HMDB_ID), compound names, formulas, monoisotopic masses, and Delta (ppm). To investigate the global metabolism variations, principal component analysis (PCA) was used to analyze all observations acquired in both ion modes. The PCA plot (Figure 4) illustrates an overview of all metabolites in the data, displaying an evident grouping trend between two groups of *A. annua* and *A. afra*. A PCA model’s quality was assessed using R^2^ (cum) as a metric, where values close to 1.0 denoted high fitness and good predictive ability. R^2^X (cum) in this study is 0.799, indicating that the PCA model developed has a sufficient level of fitness and predictive ability (Table 1).

### 2.4. Determination of the Bioactive Compounds Utilizing Supervised Pattern Recognition Analysis

**Figure 5:**
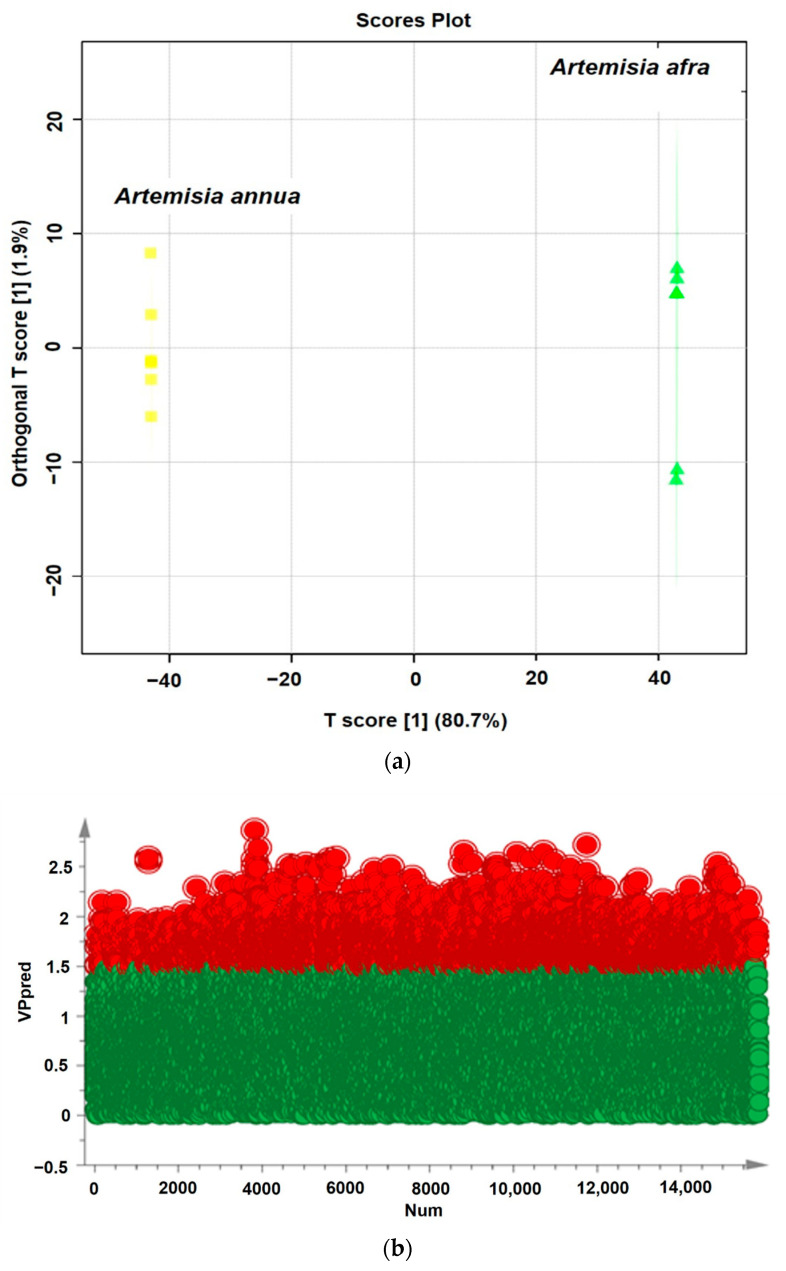

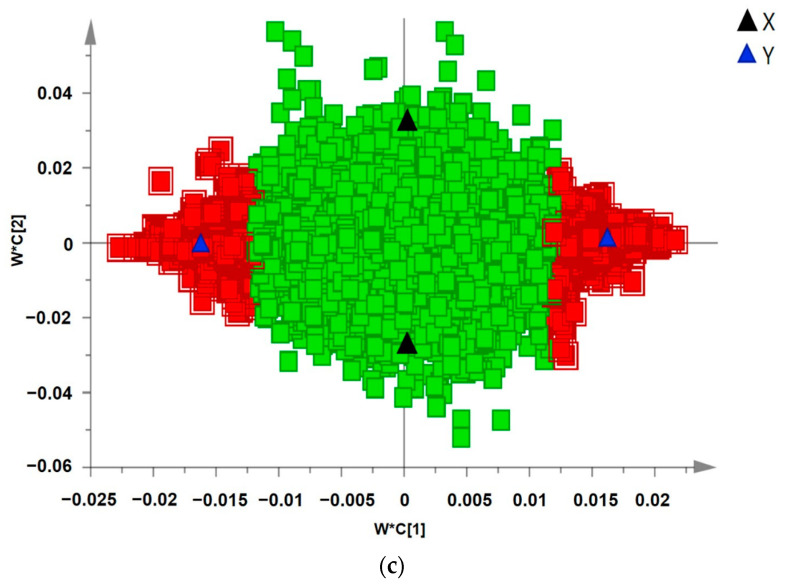
(**a**) The scores scatter plot of OPLS-DA model of *A. annua* and *A. afra* metabolites. (**b**) The distribution of VIP values (VIP > 1.5) of *A. annua* and *A. afra* metabolites. (**c**) The loading plot of PLS-DA model of *A. annua* and *A. afra* metabolites. Key: The metabolites with red color were labeled as significant compounds (VIP > 1.5) and the metabolites with green color were labeled as non-significant compounds (VIP < 1.5).

**Figure 6:**
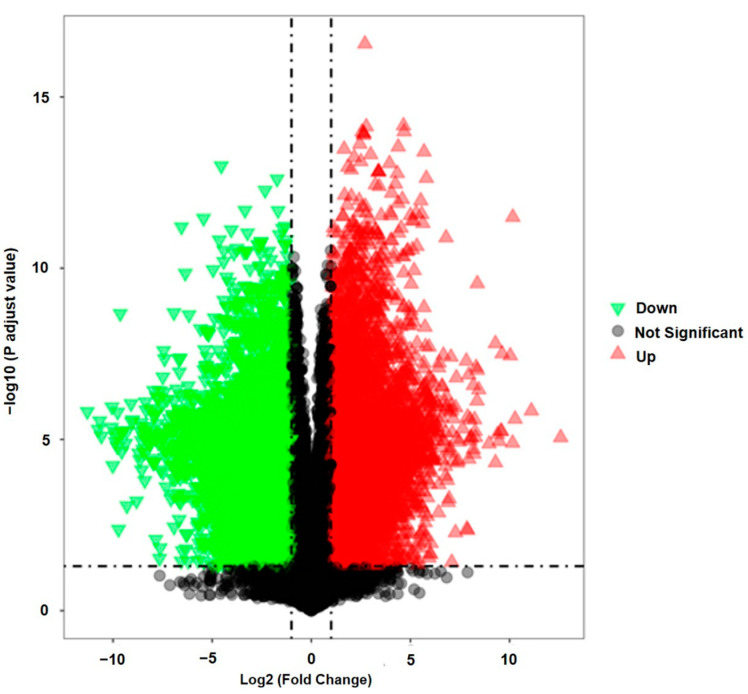
Volcano plot for group A and group B. The range of Y > 1.30 and X > 1 was a significant increase. The range of Y > 1.30 and X < −1 was a significant decrease.

**Figure 7:**
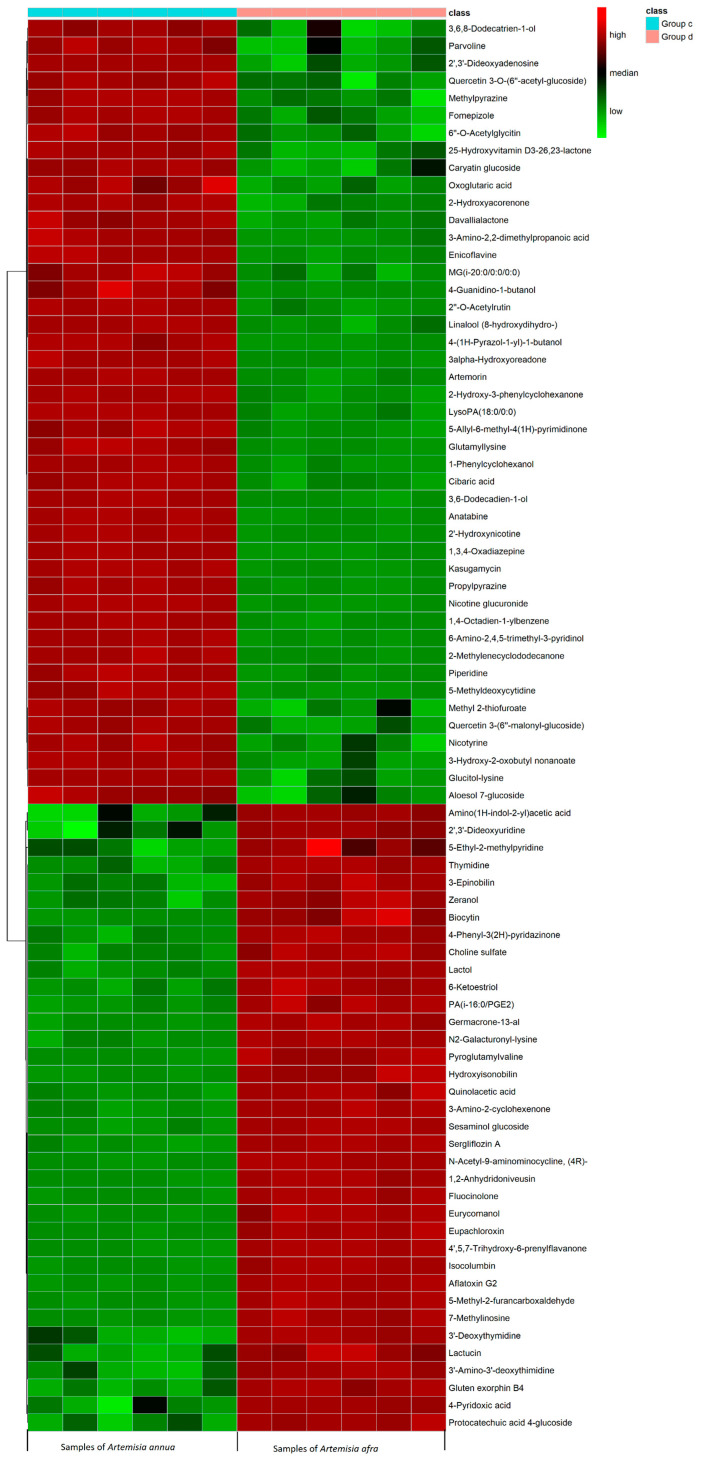
Hierarchical cluster analysis of metabolome data from group c (*A. annua)* and group d (*A. afra)* of metabolites. Hierarchical clustering analysis (HCA) was performed using the complete linkage algorithm of the program Cluster 3.0 (Stanford University) and the results are visualized using heatmap 1.0.12 (Raivo Kolde).

To eliminate any non-specific effects and predict the potential anti-COVID-19 bioactive compounds, orthogonal partial least squares discriminant analysis (OPLS-DA) and partial least squares discriminant analysis (PLS-DA) models were used to compare metabolic changes in the two groups (*A. annua* and *A. afra)*. R^2^ represents the goodness of fit, and the results indicated that the R^2^X and R^2^Y of both models were 0.793 and 1, respectively (Table 1). In the OPLS-DA score plot (Figure 5a), a clear separation of the two groups was observed. The significantly changed metabolites (ions) between the two groups were filtered out based on variable importance of projection (VIP) (VIP > 1.5), with the distribution of numerical values presented in Figure 5b. The PLS-DA loading plot shown in Figure 5c indicates the metabolites with significant compounds. The metabolites meeting the criteria of VIP > 1.5, fold change (FC) > 2.0, and *p*-value < 0.05 were selected as significant compounds. Univariate analysis, including fold change analysis and t-tests, was performed and visualized on the volcano plot (Figure 6). Table S2 (supplementary material) presents the identified potential bioactive compounds showing differences between the groups of *A. annua* and *A. afra*, under electrospray ionization ESI+ scan (positive data). Hierarchical cluster analysis supports these results, as shown in Figure 7, where a cluster of metabolome data from *A. annua* and *A. afra* groups is displayed. Metabolite ratios from two independent experiments of significant potential bioactive compounds were used for Hierarchical cluster analysis, with color intensity indicating the degree of increase (red) and decrease (green) relative to the mean metabolite ratio.

### 2.5. Binding Site Prediction of the SARS-CoV-2 Spike S Glycoprotein (6LZG)

**Figure 8:**
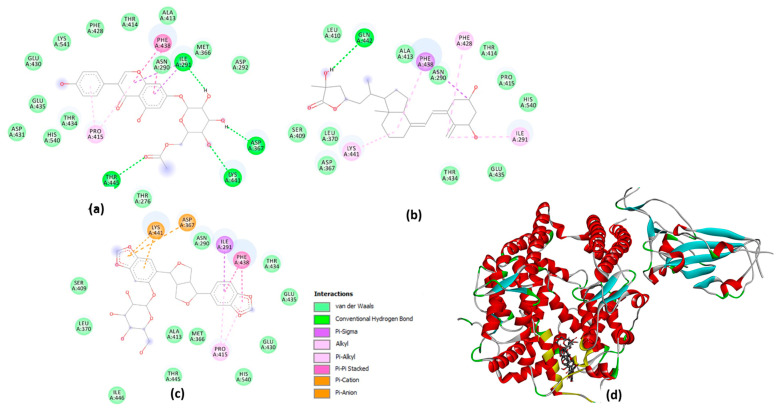
Molecular interactions plot of the three potential compounds: (**a**) 6″-O-acetylglycitin; (**b**) 25-hydroxyvitamin D3-26,23-lactone; (**c**) sesaminol glucoside with Optimal Binding Affinities against SARS-CoV-2 spike S glycoprotein. (**d**) The predicted binding site of the test compounds (stick) on the S glycoprotein spike protein.

The PrankWeb tool was employed in this study to predict and visualize the protein–ligand binding sites of the SARS-CoV-2 spike S glycoprotein (6LZG). The tool identified a pocket within the S glycoprotein highly conducive to ligand binding, demonstrated by a pocket score of 15.76 and a probability score of 0.764. The pocket score designates a specific region of the protein as a ligand-binding site, while the probability score is a value ranging from 0 to 1 that indicates the probability of the predicted pocket being a true ligand-binding site (Figure 8). This pocket, consisting of 22 amino acids, exhibited an average conservation score of 2.207. The conservation score quantifies the degree of conservation of the amino acid residues in the predicted pocket across different species, suggesting a high degree of conservation for these residues. The residues constituting this pocket were His345, Pro346, Thr347, Ala348, Asp350, Leu370, Thr371, His374, Glu375, His378, Asp382, Arg393, Asn394, Glu398, His401, Glu402, Glu406, Ser409, Leu410, Gln442, Tyr515, and Arg518.

### 2.6. Molecular Docking Studies on the Plant-Based Bioactive Compounds

The tested compounds interacted with different binding affinities with the active site of SARS-CoV-2 spike S glycoprotein (PDB code: 6LZG) and were ranked based on their binding energy (kcal/mol). Among the 81 compounds (Supplementary Material, Table S2), three compounds, namely 6″-O-acetylglycitin, 25-hydroxyvitamin D3-26,23-lactone, and sesaminol glucoside, exhibited the lowest docking scores on SARS-CoV-2 spike S glycoprotein, with binding affinities of −9.7, −9.9, and −10 kcal/mol, respectively. Consequently, they are considered promising candidates for the discovery of potential SARS-CoV-2 inhibitory drugs.

The receptor-binding domain (RBD) of the SARS-CoV-2 spike protein presents a promising target for the development of antiviral therapies. The molecular docking study successfully predicted potential compounds and elucidated the molecular interactions between the SARS-CoV-2 spike S receptor and three potential ligands: 6″-O-acetylglycitin, 25-hydroxyvitamin D3-26,23-lactone, and sesaminol glucoside.

As shown in Figure 8, all compounds similarly bind to the S receptor. The docking results indicated robust interactions of all three ligands with the receptor’s RBD domain, responsible for binding to the host cell’s angiotensin-converting enzyme 2 (ACE2) receptor. These interactions were facilitated by various non-covalent forces, including hydrogen bonds, pi-stacking, and van der Waals interactions. The specific interactions formed by the compounds with the RBD domain are as follows. 6″-O-acetylglycitin formed hydrogen bonds with the following residues: Asn290, Ile291, Asp367, Lys441, and Thr445, along with hydrophobic interactions with Pro415 and Phe438. Similarly, 25-hydroxyvitamin D3-26,23-lactone formed hydrogen bonds with Gln442 and hydrophobic interactions with ILE291, Phe428, Phe438, and Lys441. Sesaminol glucoside, exhibiting the strongest binding energy, formed hydrophobic interactions with Ile291, Pro415, and Phe438, along with electrostatic interactions with Lys441 and Asp367. The presence of these electrostatic interactions is hypothesized to account for this compound’s superior binding affinity compared to the other two compounds.

### 2.7. Molecular Dynamics Simulation Studies on the Plant-Based Bioactive Compounds

**Figure 9:**
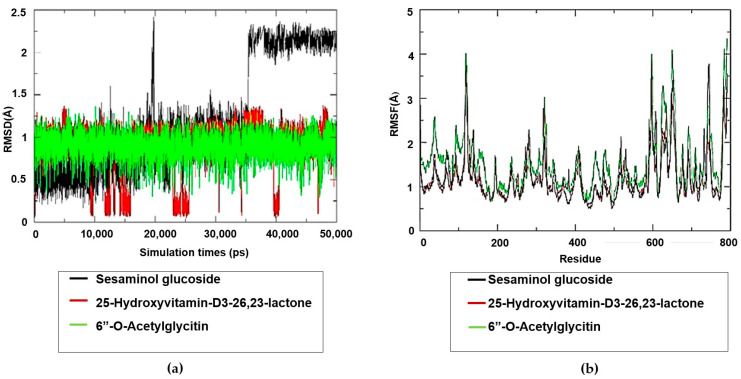
(**a**) Root mean square deviation (RMSD) plot of selected compounds with best binding affinity against spike S glycoprotein over 50 ns of molecular dynamics simulations. (**b**) Root mean square fluctuation (RMSF) plot of selected compounds with best binding affinity against spike S glycoprotein over 50 ns of molecular dynamics simulations.

The root mean square deviation (RMSD) plot for the compounds of selected ligands against spike S glycoprotein–ligand is depicted in Figure 9a. The RMSD of the 25-hydroxyvitamin-D3-26,23-lactone (red line) and 6″-O-Acetylglycitin (green line) exhibits a relatively stable average RMSD of approximately 1 Å throughout the entire simulation period (50 ns). In contrast, the RMSD of sesaminol glucoside (black line) initially remains within the range of 1 Å but significantly increases to values greater than 2 Å after 35 ns, indicating a less stable conformation compared to the two preceding compounds.

Generally, the root mean square fluctuation (RMSF) values of compounds bound to spike S glycoprotein appear to be higher, suggesting that they are less rigidly bound to spike S glycoprotein. Figure 9b depicts the RMSF values of the individual residues of the bound compounds. Typically, residues with the highest RMSF values are located in the flexible loops and protein regions prone to fluctuations, while those with the lowest RMSF values are situated in more stable regions of the proteins. However, the amino acids in spike S glycoprotein exhibit lower stability, as indicated by the RMSF of >3 Å, and are more likely to fluctuate.

### 2.8. SARS-CoV-2 Enzyme In Vitro Inhibition Assay

**Figure 10:**
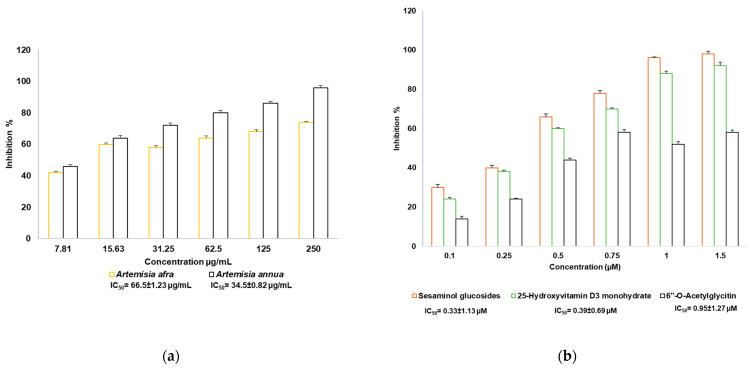
Inhibition effects of interaction between SARS-CoV-2 S1 spike protein and ACE-2 receptor by (**a**) *A. annua* and *A. afra* extracts and (**b**) compounds of 25-hydroxyvitamin D3 monohydrate, sesaminol glucoside, and 6″-O-Acetylglycitin.

The abilities of plant extracts (*A. annua* and *A. afra*) and compounds (25-hydroxyvitamin D3 monohydrate, sesaminol glucoside, and 6″-O-acetylglycitin) to inhibit the interaction of SARS-CoV-2 S1 spike protein and ACE-2 were evaluated. Both the plant extracts and all compound samples showed the potential to inhibit the interaction between SARS-CoV-2 S1 spike protein and ACE-2 receptors. However, the degree of inhibition (IC_50_) varied depending on the concentration (Figure 10). The IC_50_ values differed between the *A. annua* and *A. afra* samples. *A. annua* exhibited superior inhibition against the interaction of SARS-CoV-2 S1 spike protein and ACE-2, with the IC_50_ value of the *A. afra* sample (66.5 ± 1.23 µg/mL) being greater than that of the *A. annua* sample (34.5 ± 0.82 µg/mL). Sesaminol glucoside exhibited superior inhibition against the interaction of SARS-CoV-2 S1 spike protein and ACE-2, with an IC_50_ value of 0.33 ± 1.13 µM, followed by 25-hydroxyvitamin D3 monohydrate (0.39 ± 0.69 µM) and 6″-O-acetylglycitin (0.95 ± 1.27 µM). Comparison between the results of the in silico and in vitro study showed that 25-hydroxyvitamin D3 monohydrate exhibited a higher inhibitory effect in the in silico study, whereas 6″-O-acetylglycitin exhibited greater activity in the in vitro study. However, sesaminol glucoside consistently emerged as the most potent inhibitor in both in silico and in vitro studies.

## 3. Discussion

In 2020, the World Health Organization (WHO) welcomed innovations around the world including repurposing drugs, traditional medicines, and developing new therapies in the search for potential treatments for COVID-19 [28]. Among the medicinal plant-based bioactive compounds previously unexplored for their efficacy against COVID-19 are those from South African, *A. annua* and *A. afra*. Before conducting the SARS-CoV-2 enzyme in vitro inhibition assay, it was necessary to evaluate the cytotoxicity of the plant extracts on HEK293T cells to ensure their safety. HEK293T cells were chosen for this study because they are considered susceptible to virus proliferation [29,30]. Moreover, since HEK293 cell surfaces are not known for productive SARS-CoV-2 infections; HEK293T cells may be utilized in SARS-CoV-2 studies [31,32]. The *A. annua* and *A. afra* extracts exhibited comparable non-toxic effects on HEK293T cells at lower concentrations of 31.25, 15.63, and 7.81 µg/mL when using the resazurin cell viability assay and xCELLigence real-time cell analyzer. The real-time monitoring approach permitted label-free analysis of cell viability, providing insight into the mode of action of the test extract [33]. The xCelligence system generated cell index values as cells that were treated with the extracts. The cell index, which is influenced by the concentration of extracts, increased with decreasing concentrations of extracts administered to the cells. Records from RTCA indicating an increase in cell index values after treatment supported a proliferative phase between 24 and 98 hours when lower concentrations (31.25, 15.63 and 7.81 µg/mL) of both plant extracts were used. Several classes of phytochemicals from *A. annua* and *A. afra* involving volatile and non-volatile secondary metabolites such as artemisinin, caryophyllene oxide, caryophyllene, farnesene, germacrene D, sesquiterpenes, glaucolides, guaianolides, long-chain alkanes, organic acids, coumarins, flavonoids, and monoterpene have been identified [22,27]. Recently, there has been a surge in research on these metabolites directed to their noteworthy pharmacological activities and medicinal values [23,26]. This prompted the exploration of the metabolic profiles of several plants and the identification of valuable bioactive compounds accountable for their marked biological activities. In this study, the UPLC-MS base peak intensity (BPI) chromatograms of sample ESI+ mode of *A. annua* and *A. afra* leaf extracts exhibited a total of 225 peaks. The identified compounds belong to different classes of secondary metabolites involving triterpenoids, flavonoids, iridoid glycosides, hydroxy fatty acids, phenolic acids, carboxylic acid, phenylethanoid, and glycosides. Prior to finding the binding affinity of the identified compounds, the approach of P2Rank score as a parameter (PrankWeb) of interest was utilized, which allowed us to predict potential binding sites and assess their druggability, providing valuable insights for the development of therapeutic interventions against SARS-CoV-2. This approach offers several advantages over previous methods [34]. For instance, it not only predicts the location of potential binding sites but also provides a visual representation of these sites, which can be particularly useful for understanding the spatial relationship between the protein and potential ligands. Furthermore, the ability to compare predicted pockets with highly conserved areas and actual ligand-binding sites can provide additional insights into the functional importance of these sites [35,36]. The identified plant-based bioactive compounds were based on their highest binding affinity; 6″-O-acetylglycitin, 25-hydroxyvitamin D3-26,23-lactone, and sesaminol glucoside had the lowest docking scores on SARS-CoV-2 spike S glycoprotein, with binding affinities of −9.7 kcal/mol; −9.9 kcal/mol; and −10 kcal/mol, respectively. All identified plant-based bioactive compounds have been detected in different plant species [37,38,39]. In this study, sesaminol glucoside was found to be more abundant in *A. afra* compared to *A annua*. 6″-O-acetylglycitin and 25-hydroxyvitamin D3-26,23-lactone are abundant in *A. annua* compared to *A. afra*. Sesaminol glucoside, the compound with the strongest binding energy, formed hydrophobic interactions with Ile291, Pro415, and Phe438, as well as electrostatic interactions with Lys441 and Asp367. The presence of these electrostatic interactions is hypothesized to be the reason for this compound’s superior binding affinity compared to the other two compounds. These simulation results have significant implications for the development of antiviral therapies. The stability and rigidity of a complex may increase the likelihood that the inhibitor will prevent the protease from cleaving its target protein [40]. However, the molecular dynamics simulations conducted in this study suggest that the compounds bind less stably to S glycoprotein–ligand complexes. This stability is likely due to the fact that the S glycoprotein binding site is larger and more flexible, allowing for more ligand movement. These findings align with previous research, which found similar RMSD values for S glycoprotein–ligand complexes [41,42]. The higher RMSF values of the compounds bound to the spike S glycoprotein could be attributed to several factors, including the larger size and greater complexity of the spike S glycoprotein, its increased flexibility, and the weaker interactions between the bound compounds and the spike S glycoprotein [43,44,45]. Nonetheless, the average RMSD falls within the expected range (<3 Å) for protein complexes [46,47]. As expected, the complexes exhibiting the lowest RMSD are those with the strongest binding affinities [35].

Moreover, in the in vitro study, all compounds and plant extracts bound to the receptor’s RBD domain, which is responsible for binding to the host cell’s Angiotensin-converting enzyme II (ACE2) receptor. The binding of the SARS-CoV-2 S protein with the ACE2 of the host cell to advance membrane fusion is an important first step for SARS-CoV-2 infection. According to some researchers, such as Mani et al. [48]; Tay [49]; and Wang et al. [1], the SARS-CoV-2 spike protein interacts with the ACE2 receptor on the surface of epithelial cells from the respiratory tract or oral cavity. In the present in vitro study, the identified three best bioactive compounds and extracts from *A. annua* and *A. afra* were able to interfere with the binding between the SARS-CoV-2 spike protein and the human ACE2 receptor. Sesaminol glucoside emerged as the best inhibitor against the interaction of SARS-CoV-2 S1 spike protein and ACE-2 when compared to 6″-O-acetylglycitin and 25-hydroxyvitamin D3-26,23-lactone. Literature searches revealed limited pockets of evidence of identified bioactive compounds as blockers of SARS-CoV-2 S protein and ACE2 interaction. Natesh et al. [37] in their study of the sesaminol compound from *Sesamum indicum* recorded a weaker binding affinity of −7.0 kcal/mol against SARS-CoV-2 spike protein, forming hydrophobic interactions with different residues of spike proteins including Phe490 [50,51]. As revealed in this study, the electrostatic interactions with Lys441 and Asp367 are hypothesized to be the reason for the compound sesaminol’s superior binding affinity compared to the other two compounds. Most of the virus’s binding to ACE2 is caused by the receptor-binding motif (RBM) (437–508 amino acids) found in the RBD (319–541 amino acids) of the S1 subunit (13–685 amino acids) of the spike protein [52,53]. In observational studies, a reverse relationship was discovered between serum 25-hydroxyvitamin D (25(OH)D) concentrations and the danger or rigorousness of COVID-19 [54,55].

## 4. Materials and Methods

### 4.1. Plant Collection and Identification

The *A. annua* and *A. afra* leaves were collected from Hartbeespoort, North-West province of South Africa (25.7236° S, 27.9653° E). The identity of the plants was authenticated and confirmed by taxonomists at the National Herbarium where *A. annua* and *A. afra* sample specimens were deposited, and voucher specimens were assigned as NR 902 and NR 903, respectively. Moreover, plant collection permits have been received from the Department of Agriculture and Rural Development-Nature Conservation, South Africa (Permit No.: CF6-0234; Permit Holder: Prof. Mkolo Nqobile Monate).

### 4.2. Preparation of Plant Extracts

Five grams each of ground samples of *A. annua* and *A. afra* leaves was extracted with 50 mL of methanol (Merck, Johannesburg, South Africa) in containers placed on a Labotec shaker for 30 min and then centrifuged at 3000 rpm for approximately 15 min. The resulting extracts were filtered using Whatman No. 1 filter paper to remove plant debris, and the filtrates were allowed to dry under a stream of air at room temperature. The resulting crude extracts were dissolved in 0.5% dimethyl sulfoxide to obtain a final stock solution of 10 mg/mL. The prepared stock solutions of *A. annua* and *A. afra* leaves’ extracts were subsequently used for cell viability assays.

### 4.3. Resazurin Cell Viability Assay

The resazurin cell viability assay was conducted using HEK293T cells, purchased from Cellonex Separation Scientific, Johannesburg, South Africa. The cells were grown in Dulbecco’s Modified Eagle’s Medium (DMEM) (Sigma, Darmstadt, Germany) supplemented with 10% fetal bovine serum (FBS) (Thermo Scientific, Waltham, MA, USA). Cells were handled in a sterile laminar flow hood, which was cleaned with 70% ethanol [56].

HEK293T (1.13 × 10^6^ cells/mL) cells in media were cultivated in the incubator under a 5% CO_2_ atmosphere at 37 °C for 24 h. Thereafter, the cells were seeded at a density of 5000 cells/well in 100 µL media before treatment with plant methanol extracts of *A. annua* and *A. afra* leaves for 24 h. Then, 20 µL of resazurin dye (Sigma TOX-8) was added. The plant extracts were dissolved in 100% DMSO and further diluted with media to achieve concentrations of 250, 125, 62.5, 31.25, 15.63, and 7.81 µg/mL. A Modulus II Multifunction Plate Reader (Turner BioSystems, Sunnyvale, CA, USA) was used to measure resorufin fluorescence which was monitored at a wavelength (EM) of 590 nm, using an excitation wavelength (EX) of 560 nm. Simultaneously, absorbance readings for resorufin were recorded at 570 nm, while the peak absorbance for resazurin was observed at 600 nm wavelengths.

### 4.4. Cell Viability Assay Using xCELLigence Real-Time Cell Analyzer

In brief, the xCELLigence instrument (Real-Time Cell Analyzer—RTCA; ACEA Biosciences Inc., San Diego, CA, USA), an impedance technology with RTCA software (version 1.2.1), was utilized to monitor the exposure effects of *A. annua* and *A. afra* leaves’ methanol extracts on the viability of HEK293T cells, according to the manufacturer’s instructions [57]. The HEK293T cells were seeded at a density of 1.13 × 10^6^ cells/mL in the 96-well gold-plated E-plate and left for 24 h to adhere to the plate. Then, cells were exposed to the plant extracts with a concentration range of 250, 125, 62.5, 31.25, 15.63, and 7.81 µg/mL. Continuous real-time impedance recording of cell growth was conducted over 98 h and converted into cell index (CI) values, which were measured at 15 min intervals.

### 4.5. Metabolomics: Establishment of the Metabolites’ Bioactive Compounds

#### 4.5.1. Instruments and Reagents

Ultimate 3000LC combined with Q Exactive MS (Thermo, Waltham, MA, USA), Temp functional Centrifugation (Eppendorf, Enfield, CT, USA), ACQUITY UPLC HSS T3 (100 × 2.1 mm × 1.8 μm), Acetonitrile (Merck, Rahway, NJ, USA), methanol (Merck, Rahway, NJ, USA), formic acid (Merck, Rahway, NJ, USA), and DL-o-Chlorophenylalanine (Merck, Rahway, NJ, USA) were used.

#### 4.5.2. Sample Preparation

**Figure 11:**
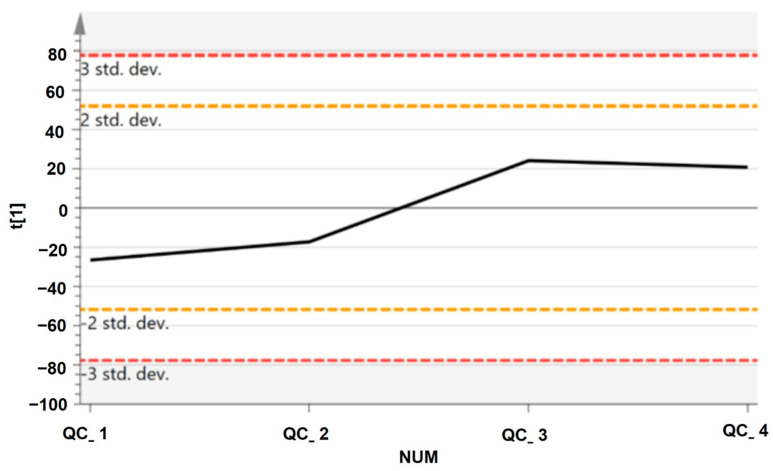
The principal component analysis (PCA) score plot of the QC samples, the X axis indicates the number of QC samples, the Y axis indicates the range of RSD.

Each sample of *A. annua* and *A. afra* leaves was lyophilized to dryness and subsequently crushed to fine powder, in a 5 mL homogenizing tube, at 30 Hz with the aid of four 5 mm metal balls on an MM 400 mill mixer. Then, 50 mg of each sample was precisely weighed into a tube and 800 μL of 80% methanol was added. Thereafter, samples were vortexed for 30 s, followed by sonication for 30 min at 4 °C. All samples were kept at −20 °C for 1 h and centrifuged at 12,000 rpm and 4 °C for 15 min. Finally, 200 μL of supernatant and 5 μL of DL-o-Chlorophenylalanine (140 μg/mL) were transferred to the vial for liquid chromatography–mass spectroscopy (LC-MS) analysis. The same amount of extract was obtained from each sample and mixed as quality control (QC) samples to evaluate the methodology. QC samples were used to demonstrate the stability of the LC-MS system. The UPLC-MS/MS raw data were acquired and aligned using Compound Discover (3.0, Thermo) based on the m/z value and the retention time of the ion signals. The ion features of the QC samples were used to calculate the relative standard deviation (RSD). The %RSD distribution is presented in Figure 11; an overwhelming majority of the RSD was less than 30%. Thus, the analysis procedure was robust and was used for subsequent sample analysis.

#### 4.5.3. Analysis of the Prepared Plant Samples Using UPLC-MS/MS

Separation was performed by Ultimate 3000LC combined with Q Exactive MS (Thermo) and screened with ESI-MS [58]. The LC system is comprised of an ACQUITY UPLC HSS T3 (100 × 2.1 mm × 1.8 μm) with Ultimate 3000LC. The mobile phase is composed of solvent A (0.05% formic acid water) and solvent B (acetonitrile) with a gradient elution (0–1 min, 95% A; 1–12 min, 95%–5% A; 12–13.5 min, 5% A; 13.5–13.6 min, 5–95% A; 13.6–16 min, 95% A). The flow rate of the mobile phase was 0.3 mL·min^−1^. The column temperature was maintained at 40 °C, and the sample manager temperature was set at 4 °C. Samples were examined in the positive ionization mode because it showed more selection and sensitivity for the LC-MS analysis of secondary metabolites in the extracts. Mass spectrometry parameters in electrospray ionization ESI+ modes are listed as follows:

ESI+: heater temp 300 °C; sheath gas flow rate, 45 arb; aux gas flow rate, 15 arb; sweep gas flow rate, 1 arb; spray voltage, 3.0 kV; capillary temp, 350 °C; S-Lens RF level, 30%.

### 4.6. Binding Site Prediction

PrankWeb, an online platform that enables the prediction and visualization of protein–ligand interaction sites, was utilized. A unique feature of PrankWeb is its ability to allow users to compare the locations of predicted pockets with areas of high conservation and actual ligand-binding sites, providing a comprehensive view of potential binding sites. We applied this method to the SARS-CoV-2 protein, specifically for protein lacking a native ligand, i.e., SARS-CoV-2 spike S glycoprotein (PDB: 6LZG). The parameter of interest was the P2Rank score, with higher values indicating a greater likelihood of the cavity being bound by ligands.

### 4.7. Molecular Docking Studies on the Bioactive Compounds

A total of 81 compounds, identified as bioactive compounds through metabolite profiling, were used as test compounds (Supplementary Material, Table S2). These compounds’ chemical structures were constructed using ChemDraw and further optimized with Chem3D, utilizing the MMFF94 force field. Concurrently, protein targets in SARS-CoV-2 were prepared by removing all non-receptor heteroatoms, including water and ions. Kollman charges were also assigned to the protein atoms. Grid boxes were positioned based on the native ligand for several proteins that possessed a native ligand. Molecular docking simulation was conducted using AutoDock Vina 1.2.3 ver 2021 based on grid dimensions for each of the protein targets, the dimensions for the SARS-CoV-2 spike S glycoprotein (PDB ID: 6LZG) were 50 Å × 50 Å × 50 Å (x = −8.974, y = −2.13, z = −26.225), with a spacing of 0.375 Å and exhaustiveness set to 8. For the protein lacking a native ligand, PrankWeb’s binding site predictions were utilized with the Probability Score set as a parameter.

### 4.8. Molecular Dynamics Simulation

The docking results with the most favorable binding energy were selected for molecular dynamics (MD) simulations [59,60]. In this study, we conducted MD simulations on the SARS-CoV-2 spike S glycoprotein (6LZG) proteins. The simulation was performed using the Amber20 package on the three compounds with the best binding affinity. During the molecular dynamics process, the protein structures were prepared using the AMBER FF99SB force field. Topology preparation files for ligands were generated using the general amber force field (GAFF) through the Antechamber module in AmberTools20. The complexes were then immersed in truncated octahedral boxes filled with TIP3P water molecules, with a buffer region of 10 Å, and neutralized by adding either Na^+^ or Cl^-^ ions. The default protonation settings in Amber20 were used for residues within the proteins. The energy minimization and MD simulations were carried out for each system utilizing the SANDER module in the Amber20 package. Approximately 10,000 cycles of minimization (MAXCYC) were performed, employing the first 500 steps of steepest descent (NCYC) and 1000 steps of conjugate gradients, addressing steric clashes and approaching an energy minimum. Following energy minimization, position restraints at constant volume (NVT) were applied for 100 ps, employing a restraint force of 10 kcal/mol at a temperature of 310 K. Subsequently, constant pressure (NPT) equilibration was conducted for 100 ps with a restraint force of 1 kcal/mol at 310 K. The next step involved equilibration under constant pressure (NPT) at 310 K, lasting 100 ps with a time step of 2 fs. During this phase, the restraint forces were removed, allowing the water to reach an equilibrium density around the protein. Langevin dynamics were employed to control temperature in both the position restraints and equilibration steps. Finally, a production run of 50 ns was performed with a time step of 2 fs under constant pressure conditions (NPT ensemble) and isotropic position scaling (ntp = 1) at 310 K.

### 4.9. SARS-CoV-2 Enzyme In Vitro Inhibition Assay

The SARS-CoV-2 Spike S1: ACE2 inhibitor screening colorimetric assay kit (Cat. No. 79954, BPS Bioscience, San Diego, CA, USA) was used to screen and profile inhibitors of this interaction according to the manufacturer’s instructions. The assay began by coating the plate with Spike S1, which was thawed on ice and diluted to 2 μg/mL in PBS. Subsequently, 50 μL of the diluted Spike S1 solution was added to each well and incubated overnight at 4 °C. After decanting the supernatant, the plate was washed three times with 100 μL 1× Immuno Buffer 1 and tapped onto clean paper towels to remove liquid. Then, each well was blocked by adding 100 μL of Blocking Buffer 2. Following a 1 h incubation at room temperature and supernatant removal, 20 μL of 1× Immuno Buffer 1 was added to each well.

Inhibitor solutions, including different plant extract concentrations (250, 125, 62.5, 31.25, 15.63, and 7.81 µg/mL) and different concentrations of 25-hydroxyvitamin D3 monohydrate (Sigma-Aldrich), Sesaminol glucoside (Sigma-Aldrich), and 6″-O-acetylglycitin (GlpBio, Monclair, NJ, USA) (1.5,1, 0.75, 0.5, 0.25, and 0.1 µM), were prepared using DMSO (0.5%). An amount of 10 μL of inhibitor solutions were added to the test inhibitor wells, while positive control and blank wells received 10 μL of inhibitor buffer (no inhibitor). ACE2-Biotin, diluted to 1 ng/μL (approximately 12 nM) in 1× Immuno Buffer 1, was added to initiate the reaction in positive control and test inhibitor wells, whereas blank wells received 20 μL of 1× Immuno Buffer 1 (Table 2).

After an hour of incubation at room temperature, the supernatant was decanted, the plate went through three washes with 100 μL 1× Immuno Buffer 1, and it was tapped onto clean paper towels to remove liquid. The wells were then blocked with 100 μL of Blocking Buffer 2 and incubated for 10 min at room temperature. Before adding 100 μL of diluted Streptavidin-HRP with Blocking Buffer 2 (1000-fold), the supernatant was removed, followed by another 1 h incubation at room temperature. The plate went through three more washes and taps onto clean paper towels to remove liquid. The wells were blocked using 100 μL of Blocking Buffer 2 and incubated at room temperature for 10 min. Thereafter, 100 μL of Colorimetric HRP substrate was added to each well, incubating until a blue color developed in the positive control wells. Finally, 100 μL of 1N HCl was added to each well, and absorbance was measured at 450 nm using a UV/Vis spectrophotometer microplate reader (Turner BioSystems, Sunnyvale, CA, USA). The tests were performed in triplicate, and percentage inhibition values for plant extracts and compounds were calculated. The results are also represented as IC_50_ ± SD.

### 4.10. Statistical Analysis

Ions from ESI+ were merged and imported into the SIMCA-P program version 14.1 (Umetrics, Umea, Sweden) for multivariate analysis. A principal component analysis (PCA) was used first as an unsupervised method for data visualization and outlier identification. Supervised regression modeling was performed on the data set using partial least squares discriminant analysis (PLS-DA) or orthogonal partial least squares discriminant analysis (OPLS-DA) to identify the potential bioactive compounds related to anti-COVID-19 activity. The bioactive compounds were filtered and confirmed by combining the results of the VIP values (VIP > 1.5) and *t*-test (*p* < 0.05). The quality of the fitting model can be explained by R^2^ and Q2 values. R^2^ displayed the variance explained in the model, indicating the quality of the fit. Q2 displayed the variance in the data, indicating the model’s predictability.

## 5. Conclusions

The findings of this study, supported by other studies, underscore the significance of integrating multiplatform metabolite profiling using UPLC-MS/MS and chemometrics to discover the chemical composition of different plant extracts. By correlating these findings with anti-COVID-19 activities, potential metabolite candidates responsible for the biological activities of the extracts can be identified. Additionally, molecular docking and in vitro studies provide a rational phytotherapeutic choice of *A. annua* and *A. afra* against SARS-CoV-2. Notably, sesaminol glucoside, 6″-O-acetylglycitin, and 25-hydroxyvitamin D3-26,23-lactone emerge as potential candidates responsible for the activities of both *A. annua* and *A. afra* extracts. Considering the findings of RMSD and RMSF values regarding the stability and rigidity of protein–ligand complexes, future research would explore the inhibitory effects of these candidates on other targets, such as 3-chymotrypsin-like proteases (3CLpro), to further understand their activities and determine their potential activities using in vitro assays.

## Figures and Tables

**Table 1:** Parameters for PCA, PLS-DA, and OPLS-DA models for *A. annua* and *A. afra* metabolite groups.

Model Type	Number of Model Dimensions	N ^(a)^	R^2^X (cum) ^(b)^	R^2^Y (cum) ^(b)^	Q^2^ (cum) ^(c)^
Parameters					
PCA-X	2	12	0.799	-	0.697
PLS-DA	2	12	0.793	1	0.999
OPLS-DA	1 + 1 + 0	12	0.793	1	0.999

Key: ^(a)^ N: number of components. ^(b)^ R^2^X (cum) and R^2^Y (cum) represent the cumulative model variations in the X and Y matrices, respectively. ^(c)^ Q^2^ (cum) represents the predictive accuracy of the model.

**Table 2:** Outline of the component’s quantities added to blank, positive control, and test inhibitor.

	Blank	Positive Control	Test Inhibitor
1× Immuno Buffer 1	40 μL	20 μL	20 μL
Inhibitor buffer (no inhibitor)	10 μL	10 μL	-
ACE2-Biotin (1 ng/μL)		20 μL	20 μL
Test Inhibitor	-	-	10 μL
Total	50 μL	50 μL	50 μL

## Data Availability

Data are included in the article or Supplementary Materials, further inquiries can be directed to the corresponding author.

## Supplementary Materials

The following supporting information can be downloaded at https://www.mdpi.com/article/10.3390/ph17070821/s1, Table S1: Distinguished metabolites in the extracts of *Artemisia annua* and *Artemisia afra* using UPLC-MS/MS set in positive ionization mode; Table S2: Identified and docked compound-based bioactive compounds from *Artemisia annua* and *Artemisia afra* (ESI+ scan).
