# Supplementary material for: Identification of South African Plant-Based Bioactive Compounds as Potential Inhibitors against the SARS-CoV-2 Receptor
Source: Pharmaceuticals (Basel). 2024 Jun 22;17(7):821. doi: 10.3390/ph17070821 (PMC11279959; doi:10.3390/ph17070821)
Supplement: Supplementary file 1 [file pharmaceuticals-17-00821-s001.zip › Table S2.pdf]

Table S2. Identified and docked compound-based bioactive compounds from *Artemisia annua* and *Artemisia afra* (ESI+ scan).

No.	Compound name	RT [min]	m/z	HMDB_ID	Formula	Monoisotopic Mass	Delta (ppm)	Log2 (FC)	T-Test	Log10 (p value)	VIP	Docked Tagert: SARS-CoV-2 spike S glycoprotein (6LZG) (kcal/mol)
1	Fomepizole	2,75	83,06094	CSID3289	C4H6N2	82,053101	7	3,50	1,59E-09	8,80	1,52	-4,2
2	Piperidine	1,227	86,09691	HMDB0034301	C5H11N	85,08914936	6	4,59	2,02E-06	5,69	1,73	-4,2
3	Methylpyrazine	2,893	95,06079	HMDB0033112	C5H6N2	94,0530982	4	5,70	3,46E-06	5,46	1,94	-4,2
4	5-Methyl-2-furancarboxaldehyde	1,024	111,04423	HMDB0033002	C6H6O2	110,0367794	2	-3,99	7,98E-07	6,10	1,62	-5,1
5	1,3,4-Oxadiazepine	2,644	138,06601	CSID67028654	C4H4N2O	96,032364	1	10,04	7,82E-08	7,11	2,56	-4,4
6	5-Ethyl-2-methylpyridine	2,804	139,12275	HMDB0029729	C8H11N	121,0891494	2	-3,98	8,79E-03	2,06	1,52	-6
7	Methyl 2-thiofuroate	0,897	143,0188	HMDB0037762	C6H6O2S	142,0088501	19	4,34	8,94E-08	7,05	1,74	-4,5
8	3-Amino-2-cyclohexenone	4,936	150,03087	CSID71227	C6H9NO	111,068413	6	-4,06	9,84E-07	6,01	1,63	-5,3
9	Parvoline	3,933	153,13835	CSID63917	C9H13N	135,104797	2	3,96	1,36E-05	4,87	1,65	-6,5
10	3-Amino-2,2-dimethylpropanoic acid	0,92	156,04193	HMDB0245805	C5H11NO2	117,0789786	1	3,63	7,72E-06	5,11	1,54	-3,7
11	Propylpyrazine	1,311	164,11804	HMDB0041571	C7H10N2	122,0843983	1	8,05	5,30E-06	5,28	2,29	-5,2
12	Oxoglutaric acid	5,003	169,01288	HMDB0000208	C5H6O5	146,0215233	13	3,55	1,29E-04	3,89	1,51	-5
13	Quinolacetic acid	2,849	169,04939	HMDB0240257	C8H8O4	168,0422587	1	-3,61	7,80E-06	5,11	1,53	-9
14	4-Phenyl-3(2H)-pyridazinone	2,954	173,07071	CSID11248873	C10H8N2O	172,06366	1	-4,82	4,03E-06	5,39	1,78	-8,3
15	4-Guanidino-1-butanol	0,787	173,13956	CSID4476579	C5H13N3O	131,105865	1	4,88	4,27E-04	3,37	1,76	-4,7
16	Nicotyrine	2,672	176,11808	HMDB0255591	C10H10N2	158,0843983	1	5,18	1,38E-05	4,86	1,86	-6,9
17	Anatabine	2,75	178,13366	CSID10910	C10H12N2	160,100052	1	8,36	8,25E-07	6,08	2,34	-7,1
18	4-(1H-Pyrazol-1-yl)-1-butanol	2,644	182,12868	CSID34452308	C7H12N2O	140,094955	1	7,00	1,09E-05	4,96	2,14	-5,1
19	Choline sulfate	0,988	184,06353	HMDB0250194	C5H13NO4S	183,0565291	1	-3,69	1,21E-05	4,92	1,55	-4
20	1,4-Octadien-1-ylbenzene	8,255	187,1479	CSID30992614	C14H18	186,140854	1	3,59	7,32E-09	8,14	1,53	-7,4
21	Amino(1H-indol-2-yl)acetic acid	3,17	191,08118	CSID32887546	C10H10N2O2	190,074234	1	-4,41	5,44E-09	8,26	1,78	-7,8

22	5-Allyl-6-methyl-4(1H)-pyrimidinone	2,32	192,11293	CSID23948557	C8H10N2O	150,079315	2	3,81	6,02E-06	5,22	1,58	-6,2
23	6-Amino-2,4,5-trimethyl-3-pyridinol	2,096	194,12868	CSID34236082	C8H12N2O	152,094955	1	8,26	3,94E-06	5,40	2,32	-6,8
24	2'-Hydroxynicotine	2,769	196,14422	HMDB0001329	C10H14N2O	178,1106131	1	11,10	1,38E-06	5,86	2,70	-6,2
25	3,6,8-Dodecatrien-1-ol	10,71	203,14697	CSID9151298	C12H20O	180,151413	12	3,60	1,02E-07	6,99	1,60	-6
26	3,6-Dodecadien-1-ol	6,947	205,15849	HMDB0031102	C12H22O	182,1670653	11	3,77	6,44E-08	7,19	1,57	-6,3
27	2-Methylenecyclododecanone	5,774	212,20058	CSID465753	C13H22O	194,167068	1	5,85	8,57E-06	5,07	1,96	-5,3
28	2',3'-Dideoxyuridine	4,976	213,09057	HMDB0245547	C9H12N2O4	212,0797069	17	-3,49	1,25E-07	6,90	1,54	-7,3
29	1-Phenylcyclohexanol	3,774	218,15369	CSID14582	C12H16O	176,120117	1	5,05	2,56E-07	6,59	1,82	-7,3
30	4-Pyridoxic acid	5,862	225,09062	HMDB0000017	C8H9NO4	183,0531578	16	-5,38	5,82E-08	7,24	1,97	-6,4
31	3'-Deoxythymidine	5,839	227,1062	HMDB0246094	C10H14N2O4	226,0953569	16	-5,95	7,73E-07	6,11	2,02	-7,2
32	Pyroglutamylvaline	5,782	229,12191	HMDB0094651	C10H16N2O4	228,111007	16	-3,47	4,94E-06	5,31	1,50	-5,5
33	2-Hydroxy-3-phenylcyclohexanone	3,169	232,13287	CSID30902651	C12H14O2	190,09938	11	6,43	1,10E-06	5,96	2,05	-8,6
34	3'-Amino-3'-deoxythimidine	4,038	242,11701	HMDB0060750	C10H15N3O4	241,106256	14	-5,09	2,30E-06	5,64	1,85	-7,2
35	Thymidine	5,406	243,10105	HMDB0000273	C10H14N2O5	242,0902716	14	-6,83	2,21E-06	5,66	2,13	-6,1
36	Germacrone-13-al	4,513	250,17963	HMDB0036881	C15H20O2	232,1463299	2	-6,74	4,10E-06	5,39	2,10	-6,4
37	2-Hydroxyacorenone	9,271	259,16643	HMDB0030916	C15H24O2	236,17763	2	4,60	4,58E-07	6,34	1,74	-5,6
38	Artemorin	7,694	266,17446	HMDB0302701	C15H20O3	248,1412445	2	6,29	9,52E-07	6,02	2,03	-7,1
39	3alpha-Hydroxyoreadone	6,425	275,12477	HMDB0036047	C14H20O4	252,1361591	2	5,16	1,95E-06	5,71	1,84	-7,3
40	2',3'-Dideoxyadenosine	6,718	277,14047	HMDB0245544	C10H13N5O2	235,1069247	1	4,34	1,56E-11	10,81	1,70	-7,1
41	5-Methyldeoxycytidine	2,036	283,13964	HMDB0002224	C10H15N3O4	241,106256	2	4,39	4,92E-06	5,31	1,69	-7,2
42	3-Hydroxy-2-oxobutyl nonanoate	6,094	286,20067	CSID67171193	C13H24O4	244,167465	3	8,00	6,42E-06	5,19	2,34	-5,5
43	Lactucin	3,633	318,13286	HMDB0035814	C15H16O5	276,0997736	2	-5,38	4,31E-04	3,37	1,88	-6,8
44	N2-Galacturonyl-lysine	6,257	323,14815	HMDB0033105	C12H22N2O8	322,1376157	10	-6,46	2,79E-06	5,55	2,06	-6,2
45	7-Methylinosine	1,031	325,139	HMDB0003950	C11H15N4O5	283,1042446	2	-6,80	7,61E-06	5,12	2,11	-6,9
46	Aflatoxin G2	6,708	331,08041	HMDB0030475	C17H14O7	330,0739528	2	-7,62	4,45E-06	5,35	2,23	-7,9
47	Protocatechuic acid 4-glucoside	2,983	339,06788	HMDB0303826	C13H16O9	316,0794321	2	-5,71	1,73E-05	4,76	1,96	-7,7
48	Glutamyllysine	2,35	339,16556	HMDB0004207	C11H21N3O5	275,1481208	5	5,59	7,30E-06	5,14	1,91	-5,9
49	6-Ketoestriol	6,526	344,18475	HMDB0000530	C18H22O4	302,1518092	3	-4,86	1,91E-05	4,72	1,78	-9

50	Glucitol-lysine	5,161	352,21098	HMDB0252764	C12H26N2O7	310,1740012	9	5,03	8,79E-09	8,06	1,83	-5,7
51	Nicotine glucuronide	1,84	356,18075	HMDB0001272	C16H22N2O6	338,1477864	2	8,28	5,38E-06	5,27	2,33	-8,8
52	4',5,7-Trihydroxy-6-prenylflavanone	3,227	358,16398	HMDB0037247	C20H20O5	340,1310737	3	-9,01	2,18E-06	5,66	2,43	-8,8
53	3-Epinobilin	3,983	364,21086	HMDB0036690	C20H26O5	346,1780239	3	-6,27	1,35E-04	3,87	2,03	-7,9
54	Zeranol	4,259	364,2109	HMDB0032702	C18H26O5	322,1780239	3	-5,67	8,31E-05	4,08	1,92	-8,3
55	Cibaric acid	4,971	366,22678	HMDB0038580	C18H28O5	324,193674	2	4,11	5,96E-07	6,22	1,64	-7
56	Isocolumbin	3,074	376,1747	HMDB0036837	C20H22O6	358,1416384	2	-8,16	7,32E-06	5,14	2,31	-8,6
57	Lactol	3,939	378,19013	HMDB0303945	C20H24O6	360,1572885	3	-7,67	7,07E-07	6,15	2,24	-3,5
58	Hydroxyisonobilin	3,359	380,20585	HMDB0034475	C20H26O6	362,1729386	2	-5,12	5,18E-05	4,29	1,82	-7,9
59	1,2-Anhydridoniveusin	1,885	394,18523	HMDB0032105	C20H24O7	376,1522031	2	-8,65	1,79E-05	4,75	2,38	-8,2
60	Kasugamycin	1,915	402,14996	CSID16736502	C14H25N3O9	379,159088	4	8,18	3,56E-07	6,45	2,31	-6,8
61	Eupachloroxin	3,586	446,1566	CSID4444792	C20H25ClO8	428,12381	2	-7,90	2,02E-05	4,69	2,27	-7,7
62	MG(i-20:0/0:0/0:0)	14,504	409,32811	HMDB0072854	C23H46O4	386,33961	2	5,86	1,59E-04	3,80	1,96	-5,6
63	Biocytin	4,469	414,21116	HMDB0003134	C16H28N4O4S	372,1831261	14	-6,26	1,38E-03	2,86	1,99	-6,8
64	Sergliflozin A	3,22	418,18493	HMDB0258246	C20H24O7	376,1522031	3	-7,79	6,77E-07	6,17	2,26	-8,3
65	N-Acetyl-9-aminominocycline, (4R)-	4,66	420,20061	HMDB0259457	C20H31NO7	397,2100523	3	-9,55	4,18E-06	5,38	2,50	-8,4
66	Enicoflavine	3,338	423,17518	CSID4444887	C10H13NO4	211,084457	2	3,64	2,11E-06	5,67	1,54	-5,3
67	Eurycomanol	1,378	428,19056	HMDB0252130	C20H26O9	410,1576824	2	-5,56	1,18E-05	4,93	1,90	-7,7
68	Aloesol 7-glucoside	4,058	438,17503	HMDB0040565	C19H24O9	396,1420324	2	3,43	2,49E-07	6,60	1,51	-9,4
69	25-Hydroxyvitamin D3-26,23-lactone	11,375	451,28331	HMDB0060126	C27H40O4	428,2926598	3	6,77	1,30E-06	5,89	2,13	-9,9
70	Fluocinolone	2,966	454,20623	HMDB0252347	C21H26F2O6	412,1697449	6	-8,07	3,77E-06	5,42	2,30	-8,2
71	LysoPA(18:0/0:0)	10,568	461,26759	HMDB0007854	C21H43O7P	438,2746407	8	5,34	4,21E-08	7,38	1,87	-5,6
72	Davallialactone	5,955	465,11703	HMDB0250883	C25H20O9	464,1107322	2	4,28	1,62E-05	4,79	1,67	-9,6
73	6''-O-Acetylglycitin	5,524	489,13817	HMDB0039489	C24H24O11	488,1318616	2	4,53	1,14E-05	4,94	1,73	-9,7
74	Quercetin 3-O-(6''-acetyl-glucoside)	3,718	507,11242	HMDB0029271	C23H22O13	506,1060408	2	3,46	9,13E-07	6,04	1,50	-8,6
75	Caryatin glucoside	4,025	529,13309	HMDB0037352	C24H26O12	506,1424263	3	4,13	3,72E-06	5,43	1,67	-8,7
76	Gluten exorphin B4	6,575	547,21614	HMDB0059794	C24H27N5O9	529,1808775	3	-5,58	2,07E-08	7,68	1,93	-8,4
77	Quercetin 3-(6''-malonyl-glucoside)	4,937	551,10198	HMDB0037368	C24H22O15	550,09587	2	6,00	7,96E-06	5,10	2,00	-8,9

78	Sesaminol glucoside	7,259	555,14816	HMDB0041209	C26H28O12	532,1580764	2	-6,32	5,81E-07	6,24	2,03	-10,3
79	2''-O-Acetylrutin	8,193	691,12656	HMDB0039929	C29H32O17	652,1639496	1	4,94	2,19E-07	6,66	1,80	-9,2
80	Linalool (8-hydroxydihydro-)	4,627	692,28835	HMDB0304700	C32H42O14	650,257456	4	5,32	4,52E-10	9,34	1,87	-6,1
81	PA(i-16:0/PGE2)	12,248	808,46495	HMDB0267751	C39H69O11P	744,45775	11	-5,37	4,23E-05	4,37	1,87	***

Key: Compound 81*** cannot be docked due to the silicon "Si" atom in the structure. Green highlight represents top three compounds with the lowest binding energy which have best affinities.
